# Serum cobalt and chromium concentration following total hip arthroplasty: a Bayesian network meta-analysis

**DOI:** 10.1038/s41598-023-34177-w

**Published:** 2023-04-27

**Authors:** Filippo Migliorini, Marco Pilone, Andreas Bell, Ricarda Merfort, Riccardo Giorgino, Nicola Maffulli

**Affiliations:** 1 Department of Orthopaedic, Trauma, and Reconstructive Surgery, RWTH University Hospital, Pauwelsstraße 30, 52074 Aachen, Germany; 2 Department of Orthopaedic and Trauma Surgery, Eifelklinik St.Brigida, 52152 Simmerath, Germany; 3 Department of Medicine, Surgery and Dentistry, University of Salerno, 84081 Baronissi, SA Italy; 4 Residency Program in Orthopedics and Traumatology, University of Milan, Milan, Italy; 5 School of Pharmacy and Bioengineering, Faculty of Medicine, Keele University, ST4 7QB Stoke On Trent, England; 6 Barts and the London School of Medicine and Dentistry, Centre for Sports and Exercise Medicine, Queen Mary University of London, Mile End Hospital, E1 4DG London, England

**Keywords:** Medical research, Signs and symptoms, Materials science

## Abstract

The present systematic review investigated the concentration of chromium (Cr) and cobalt (Co) in serum in patients who have undergone total hip arthroplasty (THA). The first outcome of interest was to investigate the mean concentration in serum of Cr and Co using different material combinations and to verify whether their concentrations change significantly using different patterns of head and liner in THA. The second outcome of interest was to investigate whether the time elapsed from the index surgery to the follow-up, BMI, sex, and side exert an influence on the mean concentration of Cr and Co in serum in patients who have undergone THA. The following material combinations were investigated (head-liner): Ceramic-Co Cr (CoCr), CoCr-CoCr, CoCr-Polyethylene, CoCr high carbide-CoCr high carbide. Data from 2756 procedures were retrieved. The mean length of follow-up was 69.3 ± 47.7 months. The ANOVA test evidenced good comparability in age, length of follow-up, BMI, and sex (P > 0.1). In patients who have undergone THA, the mean concentration in the serum of Co ranged between 0.5 µg/L and 3.5 µg/L, and the mean concentration of Cr from 0.6 to 2.6 µg/L. The difference in the concentration of Co and Cr in serum is strictly related to the implant configuration, with the coupling CoCr-CoCr showing the highest and CoCr-Polyethylene showing the lowest concentration. Patient characteristics, BMI, sex, side and the time elapsed from the index surgery to the last follow-up did not exert a significant influence on the concentration of Co and Cr in serum in patients who have undergone total hip arthroplasty (THA).

## Introduction

Total hip arthroplasty (THA) is a common procedure for patients with hip osteoarthritis. THA is associated with a significant improvement in patient reported outcome measures (PROMs)^1–3^. The weight bearing on the mobile components (head and liner) of THA produce friction, wear, tear, and deformation, and consequently the release of metal elements^4^. Particles release in implants with metallic mobile components, especially chromium (Cr) and cobalt (Co), is a concern^1,5^. These particles might remain into the joint capsule or migrate to the periarticular tissues or to other body sites though the blood and lymphatic circulation. The concentrations of Co and Cr in patients who have undergone THA with Co-Cr components are detectable in their serum. Several studies have been conducted to assess the serum concentration of Co and Cr in patients with such mobile components^4,6–10^. However, variability in implant components may impair a proper estimation of the serum concentration. Whether different mobile component configurations in THA (Ceramic-CoCr, CoCr-CoCr, CoCr-Polyethylene) is associated with differences in serum concentrations of Co and Cr is unclear and evidence is missing. Moreover, whether patient demographic may influence the serum concentration of Co and Cr has not been systematically evaluated. Recently, Co-Cr alloys have been enhanced with high carbide alloy (Co-Cr_HC_) additives to increase the stability of the metals, and therefore, reduce wear, tear, and deformation over the time^11–13^. However, whether Co-Cr_HC_ is associated with a lower concentration of Co and Cr is also unclear.

The present systematic review investigated the concentration of Co and Cr in the serum of patients who had undergone THA. The first outcome of interest was to investigate the mean serum concentration of Cr and Co in patients who have undergone THA using different material combinations, and to verify whether their concentrations change significantly using different head and liner coupling. The second outcome of interest was to investigate whether the time elapsed from the index surgery to the follow-up, BMI, sex, and side exert an influence in the mean concentration in serum of Cr and Co. The following material combinations were investigated (head- liner): Ceramic-CoCr, CoCr-CoCr, CoCr-Polyethylene, CoCr_HC_-CoCr_HC_. It was hypothesised that patient characteristics and the time elapsed from the index surgery to the last follow-up did not exert a significant influence on the concentration of Co and Cr in serum.

## Methods

### Eligibility criteria

All the clinical trials investigating the concentration (µg/L) of Cr and/ or Co in serum in patients who have undergone THA were considered. Only studies which clearly stated the composition of head and/ or liner components were eligible. Reviews, opinions, letters, editorials were not considered. In vitro, computational, biomechanics, and animal studies were not eligible. Prospective studies level I to II of evidence, according to Oxford Centre of Evidence-Based Medicine^14^, were considered. Given the authors language abilities, articles in English, German, Italian, French and Spanish were eligible. Missing data on the mean serum concentration (µg/L) of Cr and Co warranted the exclusion from the present study.

### Search strategy

This study compiles with the Preferred Reporting Items for Systematic Reviews and Meta-Analyses: the 2020 PRISMA checklist^15^. The PICOTD algorithm was preliminary pointed out:

- P (Problem): end-stage OA;
- I (Intervention): THA;
- C (Comparison): Ceramic-CoCr, CoCr-CoCr, CoCr-Polyethylene, CoCr_HC_-CoCr_HC_;
- (Outcomes): concentration in serum;
- T (Time): minimum 24 months follow-up;
- D (Design): clinical trial.

In December 2022, the following databases were accessed: PubMed, Web of Science, Google Scholar, Embase. No time constrain was set for the search. The following matrix of keywords were used in each database to accomplish the search using the Boolean operator AND/OR: THA AND (OR hip OR arthroplasty OR replacement OR prosthesis) AND (serum OR blood OR plasma) AND (CoCr OR Cr Co OR Cr OR Co OR metal OR steel OR high carbide). No additional filters were used in the databases search.

### Selection and data collection

Two authors (F. M. and R.M.) separately performed selection and data collection. The full-text of the studies which matched the topic of interest were accessed. If the full-text was not, the article was excluded. The references of the full-text articles were screened by hand by the reviewers for inclusion. In case of disagreements, a third author (N.M.) took the final decision.

### Data extraction

Two authors (F.M. and R.M.) independently performed data extraction in a Microsoft Office Excel spreadsheet (version 16, Microsoft Corporation, Redmond, USA). The following generalities were retrieved: first author, year, length of the follow-up, and journal of publication. The following data at baseline were collected: number of patients, women, side, mean age, and mean BMI (Kg/m^2^). Data concerning the mean serum concentration (µg/L) of Cr and Co were extracted at last follow-up.

### Assessment of the risk of bias

The risk of bias was evaluated in accordance with the guidelines in the Cochrane Handbook for Systematic Reviews of Interventions^16^. Two reviewers (R.G. and A.B.) evaluated the risk of bias of the extracted studies independently using the risk of bias of the software Review Manager 5.3 (The Nordic Cochrane Collaboration, Copenhagen). The following endpoints were evaluated: selection, detection, performance, attrition, reporting, and other bias. Disagreements were solved by a third author (N.M.).

### Synthesis methods

The statistical analyses were performed by the main author (F.M.) following the recommendations of the Cochrane Handbook for Systematic Reviews of Interventions^17^. For descriptive statistics, mean and standard deviation were used. To evaluate baseline comparability of patient demographic, the SPSS software was used. The analysis of variance (ANOVA) was performed assuming that values of P > 0.05 indicated comparability. The STATA/MP software (Stata Corporation, College Station, Texas, USA) was used for the network meta-analysis. The analyses were performed through the STATA routine for Bayesian hierarchical random-effects model. Continuous variables were analysed through the inverse variance method with standardized mean difference (SMD) effect measure. The confidence interval was set at 0.95. Heterogeneity was assessed using χ^2^ and Higgins-I^2^ tests. If χ^2^ > 0.05, no statistically significant heterogeneity was found. A fixed model effect was used. If χ^2^ < 0.05 and Higgins-I^2^ > 60% high heterogeneity was found and a random model effect was used for analysis. A multiple linear model regression analysis through the Pearson Product-Moment Correlation Coefficient (r) was used. The Cauchy–Schwarz formula was used for inequality: + 1 is considered as positive linear correlation, while and − 1 a negative one. Values of 0.1 <| r |< 0.3, 0.3 <| r |< 0.5, and | r |> 0.5 were considered to have weak, moderate, and strong correlation, respectively. The overall significance was assessed through the χ^2^ test, with values of P < 0.05 considered statistically significant.

### Ethical approval

This study complies with ethical standards.

## Results

### Study selection

**Figure 1:**
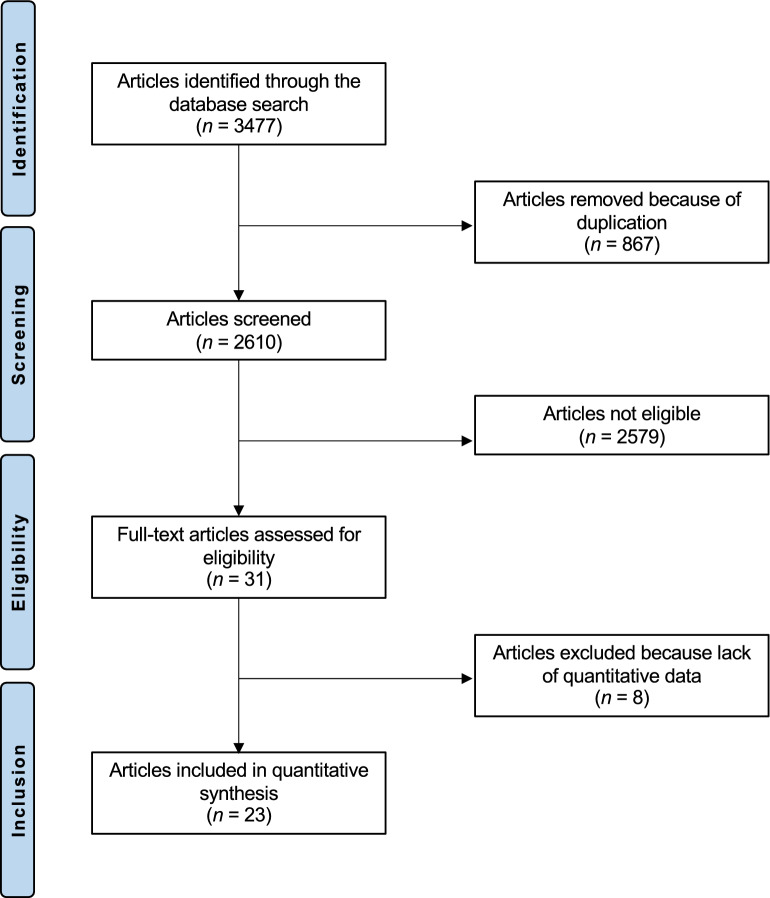
PRISMA flow chart of the literature search.

The initial databases research resulted in 3477 articles. Of them 867 were excluded as they were duplicates. A further 2579 articles were excluded as they did not match the eligibility criteria: not reporting data on the concentration in Co and/ or Cr in serum (N = 1733), study design (N = 385), not focusing on THA (N = 329), poor level of evidence (N = 84), not clearly reported the composition of head and/ or liner (N = 45), language limitations (N = 3). A further eight studies were excluded as they did not report quantitative data under the outcomes of interests. Finally, 23 studies were included: 15 nonRCTs and 8 RCTs. The results of the literature search are shown in Fig. 1.

### Risk of bias assessment

**Figure 2:**
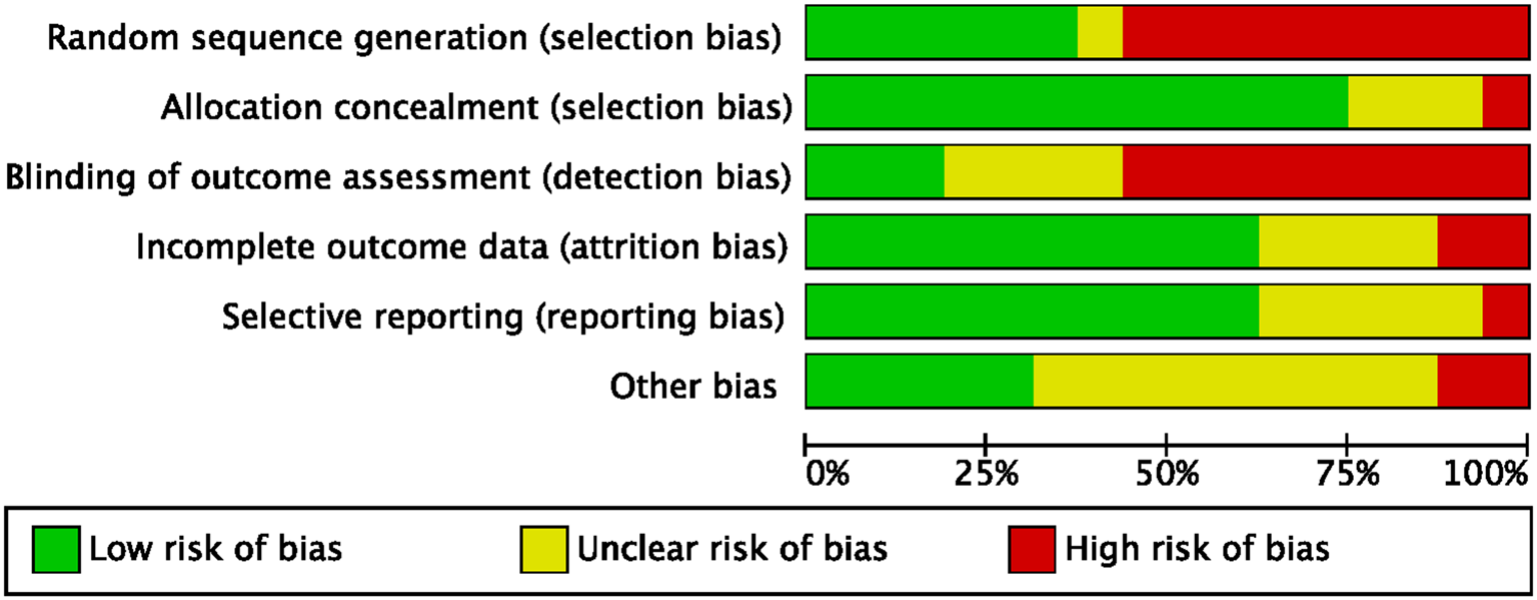
Cochrane risk of bias tool.

The risk of bias tool of the Cochrane Collaboration was used to evaluate the risk of bias. Given the prospective nature of the included studies, the overall risk of selection bias was low to moderate. Most studies did not perform assessor blinging or gave no information on it. Therefore, the risk of detection bias was moderate to high. The overall risk of attrition and reporting biases were both low to moderate, and the risk of other bias was moderate. Concluding, the overall quality of the methodological assessment was low to moderate (Fig. 2).

### Study characteristics and results of individual studies

Data from 2756 THAs were retrieved. Of them, 53% (1461 of 2756) were performed on women. The mean length of the follow-up was 69.3 ± 47.7 months. The mean age was 59.9 ± 8.6 years and the mean BMI was 28.2 ± 1.9 kg/m^2^. The ANOVA test evidenced good comparability in age, follow-up, BMI and sex of the patient demographic (P > 0.1). The generalities of the included studies are shown in Table 1, and the patient demographic of each group is shown in Table 2.

**Table 1:** Generalities and patient baseline of the included studies.

Author and year	Design	Head material	Liner material	Procedures	Mean age	Mean BMI	Women (%)
Briggs et al. 2015^9^	NonRCT	CoCr	Polyethylene	22	73	28.7	77%
		Cocr	CoCr	23	67	28.1	74%
Cadossi et al. 2016^18^	NonRCT	Ceramic	CoCr	20	65.9	28.5	70%
		CoCr	CoCr	29	61.8	26	41%
Chen et al. 2016^8^	NonRCT	CoCr_HC_	CoCr_HC_	25	36		
		CoCr	Polyethylene	25	35.4		
Dahlstrand et al. 2017^10^	RCT	CoCr	CoCr	41	65	27	51%
		CoCr	Polyethylene	44	67	27	54%
Darrith et al. 2020^19^	NonRCT	CoCr	CoCr	49	57.59	33.58	51%
		Al^2^O^3^, CoCr	CoCr, ceramic, polyethylene	26	58.65	33.72	50%
Engh et al. 2014^20^	RCT	CoCr	Polyethylene	33	61.6	29.9	30%
		CoCr	CoCr	22	62.2	28.7	60%
		CoCr	CoCr	30	63.4	29.1	29%
		CoCr	CoCr	30	63.4	29.1	29%
Grübl et al. 2006^21^	RCT	Al^2^O^3^	Al^2^O^3^	15	58.2	28.2	53%
		CoCr	CoCr	13	66.8	28.2	77%
		Al^2^O^3^	Al^2^O^3^	15	58.2	28.2	53%
		CoCr	CoCr	13	66.8	28.2	77%
Gustafson et al. 2014^22^	RCT	CoCr	CoCr	19	64	26	53%
		Al^2^O^3^, CoCr	Polyethylene	25	64	27	72%
		CoCr	CoCr	19	64	26	53%
		Al^2^O^3^, CoCr	Polyethylene	25	64	27	72%
Higgins et al. 2020^23^	RCT	CoCr	CoCr	87	65.2		37%
		AMC/ZTA	CoCr	92	65.2		37%
Malviya et al. 2011^24^	RCT	CoCr	CoCr	50	63.9	28.6	62%
		CoCr	Polyethylene	50	64.9	29.4	54%
		CoCr	CoCr	50	63.9	28.6	62%
		CoCr	Polyethylene	50	64.9	29.4	54%
Martin et al. 2018^25^	NonRCT	AMC/ZTA	AMZ/ZTA	42	60	26.4	14%
		CoCr	CoCr	40	54	30.6	55%
Moroni et al. 2012^26^	NonRCT	CoCr	PCU	15	67	27.7	60%
		CoCr	CoCr	15	61	25.5	60%
		CoCr	PCU	15	67	27.7	60%
		CoCr	CoCr	15	61	25.5	60%
Nam et al. 2015^27^	NonRCT	CoCr	Polyethylene	10	54.2	27.3	50%
		Ceramic	Polyethylene	15	45.1	26	80%
		OxZr	Polyethylene	11	43.5	30.3	36%
Pozzuoli et al. 2020^28^	NonRCT	CoCr	CoCr	34	66.1	24.3	68%
		Ceramic	AMZ/ZTA	34	68.6	25.5	62%
Savarino et al. 2008^29^	NonRCT	CoCr	CoCr	32	72		75%
		Al^2^O^3^	Al^2^O^3^	16	54		56%
		Control group	Control group	47	43		21%
Savarino et al. 2002^30^	NonRCT	CoCr	CoCr	26	48		54%
		CoCr	Polyethylene	15	64		80%
		Control group	Control group	22	56		59%
		Control group	Control group	22	43		36%
Savarino et al. 2008^29^	NonRCT	CoCr	CoCr	32	72		75%
		Al^2^O^3^	Al^2^O^3^	16	54		56%
		Control group	Control group	47	43		21%
Savarino et al. 2002^30^	NonRCT	CoCr	CoCr	26	48		54%
		CoCr	Polyethylene	15	64		80%
		Control group	Control group	22	56		59%
		Control group	Control group	22	43		36%
Schouten et al. 2017^31^	NonRCT	AMC/ZTA	CoCr	36	62	30	50%
		CoCr	CoCr	31	64	30	32%
Schouten et al. 2012^32^	RCT	AMC/ZTA	CoCr	41	61.5	29	45%
		CoCr	CoCr	36	63.8	29	36%
		AMC/ZTA	CoCr	41	61.5	29	45%
		CoCr	CoCr	36	63.8	29	36%
Tiusanen et al. 2013^33^	NonRCT	CoCr_HC_	CoCr_HC_	46	62		50%
		CoCr_HC_	Polyethylene	46	60		48%
		CoCr_HC_	CoCr_HC_	46	62		50%
		CoCr_HC_	Polyethylene	46	60		48%
White et al. 2016^34^	NonRCT	AMC/ZTA	Polyethylene	370	60.6	27.5	43%
		CoCr	Polyethylene	313	74.2	27.2	60%
Zijlstra et al. 2014^35^	RCT	CoCr	Polyethylene	32			
		CoCr	CoCr	28			
		CoCr	Polyethylene	32			
		CoCr	CoCr	28			

*RCT* randomised controlled trial, *Al*^*2*^*O*^*3*^ Alumina oxide ceramic, *AMC/ZTA* Alumina matrix composite/Zirconia toughed alumina, *OxZR* Oxidized zirconium, *PCU* Polycarbidate Urethan, *CoCR* Co Cr, *CoCR*_*HC*_ CoCr—high carbid.

**Table 2:** Demographic of the patients of each group (CoCR: Co Cr; CoCR_HC_: CoCr—high carbid).

Materials (head-liner)	THAs	Mean age	Mean BMI	Women
Ceramic-CoCr	230	63.2 ± 2.2	29.1 ± 0.6	49%
CoCr-CoCr	981	62.3 ± 5.5	28.1 ± 2.1	52%
CoCr-polyethylene	811	63.0 ± 9.8	27.7 ± 2.4	57%
CoCr_HC_-CoCr_HC_	258	59.6 ± 11.0	28.0 ± 1.4	45%
Control group	232	50.4 ± 26.4	26.4 ± 3.8	35%

### Mean concentration of Co and Cr in serum

The mean concentration of Co in serum ranged between 0.5 µg/L and 3.5 µg/L. The mean concentration of Cr in serum ranged between 0.6 and 2.6 µg/L. The concentration of both materials according to the different head- liner compositions is shown in Table 3.

**Table 3:** Mean concentration in serum of Co and Cr using different materials combination.

Materials	Co (µg/L)	Cr (µg/L)
Ceramic-CoCr	1.7 ± 1.0	1.3 ± 0.6
CoCr-CoCr	3.5 ± 5.1	2.6 ± 4.4
CoCr-Polyethylene	0.5 ± 0.5	0.6 ± 0.4
CoCr_HC_-CoCr_HC_	0.7 ± 1.1	1.1 ± 1.7
Control Group	0.3 ± 0.1	0.3 ± 0.2

### Chromium

**Figure 3:**
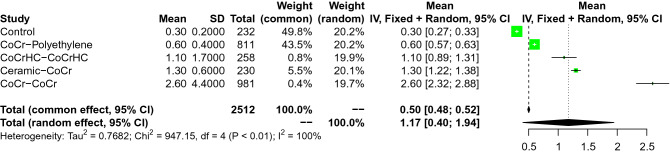
Forest plot of the comparison on Cr.

The coupling CoCr-Polyethylene demonstrated the lowest concentration of Cr in serum, followed by CoCh_HC_-CoCh_HC_, and Ceramic-CoCr. The coupling CoCr-CoCr demonstrated the highest concentration of Cr in serum. The overall effect was significant (95% CI: 0.0781 to 0.1225, Fig. 3). All network comparisons are showed in Appendix A.

### Cobalt

As expected, the control group and the coupling CoCr-CoCr demonstrated the lowest and the highest concentration of Cr in serum, respectively. After the control group, the coupling CoCr-Polyethylene demonstrated the lowest concentration of Cr in serum, followed by the coupling CoCh_HC_-CoCh_HC_, Ceramic-CoCr. The overall effect was significant (95% CI 0. 0.1345–0.1871, Fig. 4). All network comparisons are showed in Appendix B.

**Figure 4:**
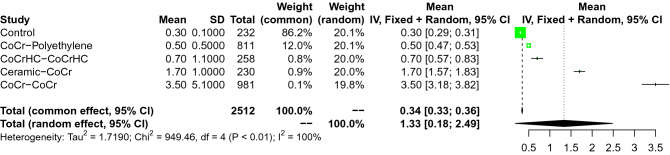
Forest plot of the comparison on Co.

### Multiple linear regressions

There was evidence of a weak association between BMI and the concentration of Co in serum (r = 0.3; P = 0.03). The time elapsed from the index surgery to the last follow-up, sex, and side did not evidence any statistically significant association with the concentration of Co and Cr in serum (Table 4).

**Table 4:** Multiple linear regressions.

Endpoint	Co		Cr	
	*r*	P	*r*	P
Follow-up	0.1	0.9	0.1	0.9
BMI	0.3	0.03	0.2	0.2
Male/female	0.6	0.6	0.1	0.9
Right/left	0.3	0.3	0.2	0.2

## Discussion

According to the main findings of the present study, the mean concentration of Co in the serum of patients who have undergone THA ranged between 0.5 µg/L and 3.5 µg/L, and the mean concentration of Cr from 0.6 to 2.6 µg/L. The difference in the concentration of Co and Cr in serum is strictly related to the implant configuration, with the coupling CoCr-CoCr showing the highest and the coupling CoCr-Polyethylene showing the lowest concentration. These results confirm our hypothesis that patient characteristics and the time elapsed from the index surgery to the last follow-up did not exert any significant influence on the concentration of Co and Cr in serum in patients who have undergone THA.

Co exists in two forms: Co^2+^ and Co^3+^ and the absorption is mediated by the same receptor of Fe^2+^^36,37^. Co has an important role as a constituent of vitamin B 12 (hydroxocobalamin)^38^. Occupational exposure to Co typically happens in hard metal industry, with the inhalation of dust; in the construction industry, through skin contact with cement; in the e-waste recycling industry, from the release of Co from several electronic devices^36,39,40^. Co can be toxic for different organs due to the accumulation and the oxidative stress^36^. Co can cause a rapid and reversible decline of cardiac systolic function^41^. Co can cross the blood–brain barrier and cause peripheral and central nervous system deficit^42^. Hearing loss, optic nerve atrophy, cognitive decline, motor axonopathy, and sensitive symptoms have been documented^43–45^. Co inhalation is associated with the ‘hard metal lung disease’^46^. Skin contact provokes contact dermatitis and it is considered an occupational disease ^47^. The hematologic effect of Co is uncertain: some studies show an association between red blood cell count and haemoglobin levels and Co concentration^48,49^. Co decreases the iodine uptake by the thyroid resulting in gout and the development of hypothyroidism^50^. Exposure to Co, associated with tungsten carbide (WC–CO) can augment the risk of developing lung cancer^51,52^. The WC–CO nanoparticles generate ROS and promote cells proliferation and inflammation^53^.

Cr exists in different oxidation states from − 2 to + 6^54^. Cr enters the cells through specific transporters, and it is reduced by glutathione reductase^55^. During this process, several reactive oxygen species can be formed, including ion superoxide and hydrogen peroxide^55^. Cr is excreted by the kidneys and through bile and hair in lower proportion^56^. Cr hazard has spread given its industrial usage^57^. Because of the heavy water contamination, urban areas are more at risk than rural areas ^58^. The established threshold of Cr in drinking water is 0.1 mg/l^59^. Inhalation of Cr can cause parenchymal pneumonia, asthma, wheezing and mucosal lung damage^60,61^. Gastrointestinal symptoms of Cr ingestion are bloody diarrhoea, abdominal pain, vomiting and ulceration^62^. High concentration of Cr can provoke hepatotoxicity, causing necrosis of liver cells and lymphocytes infiltration, leading to liver dysfunction^55,63^. Cr has toxic effects on the reproductive system^64,65^. Cr induces an increase in IGF-1 receptors, FOXO1 and an elevation in p53 expression level in kidney cells^66^. Chronic exposure can cause tubular necrosis and renal failure^66^. Contact dermatitis is common among workers in leather factories, and it is classified as an occupational disease^67^. Cr is an extremely sensitizing agent, both through inhalation and skin contact^67,68^. Cr is a genotoxic agent and is carcinogen^69^. Professional exposure can cause lung and sinonasal cancer^70^. It can also be related with gastrointestinal tract cancer^71^.

Adverse reaction to metal debris (ARMD) was described after metal-on-metal (MOM) THA, caused by the corrosion of the head and neck component^72^. Metal particles induce a local inflammatory reaction that can provokes fibrosis and osteolysis^36^. ARMD includes different histological findings^73^. In metallosis, the activation of innate response induces the formation of a granuloma surrounding metal debris^74^. Aseptic lymphocytic vasculitis associated lesion is characterized by perivascular lymphocytic infiltration and lymphoid aggregates of B and T cells, similar to a type IV reaction^75^. Type I reaction is mediated by immunoglobulin^76^. Radiography is the first line investigation for the diagnosis although it is not sensitive (62–64%)^73^. Periprosthetic osteolysis or a radiodense joint effusion can be identified^77–79^. MRI is the most sensitive imaging to diagnose ARMD^77^. It can detect indirect signs such as wear-induced synovitis, and direct signs generated by magnetic field variation, produced by metal fragments^80–82^.

Our systematic review includes the most updated articles in the present literature. 8 RCT studies were included in this review. The other studies had an overall low-moderate risk of bias. This makes our conclusion very reliable. Our study did not examine only one type of implant, but it compared ions concentrations using different materials patterns. It allows the surgeon to have a comprehensive understanding of the risks of ions related diseases when a specific type of implant is chosen. To our knowledge, this is the first systematic review that examined the association between ions concentration and the patients’ characteristics. This is another step ahead for the personalised surgery.

The present study has limitations. Firstly, the retrospective nature of some studies included in our review. Patient selection was different among the included studies. Patients suffering from renal failure were not excluded in studies^8,10,20,23–26,29,30^. The predominant mechanism of Cr and Co excretion is glomerular filtration without reabsorption^54,83^. Renal failure can lead to an accumulation of the two ions and an increase in their toxicity, but no association was found between GFR and ion levels^84,85^. It is not clear whether renal failure is a contraindication for metal-on-metal implants, but in these patients, a strict follow-up is advised^21,86,87^. It could influence the ion concentration values. It was not used a standardised method for blood sample collection. Pre-operative data were not available in two studies^19,28^. Country, region, city closeness to the factory, pollution of the ground and even the season can influence ion levels in the blood serum^88^. The diameter of the femoral head implant was not well clarified among the included studies. It is shown that a femoral head diameter greater than 36 mm is correlated with ARMD^33,89,90^.

## Conclusion

The mean concentration of Co in the serum of patients who have undergone THA ranged between 0.5 µg/L and 3.5 µg/L, and the mean concentration of Cr from 0.6 to 2.6 µg/L. The difference in the concentration of Co and Cr in serum is strictly related to the implant configuration, with the coupling CoCr-CoCr showing the highest and the coupling CoCr-Polyethylene showing the lowest concentration. Patient characteristics and the time elapsed from the index surgery to the last follow-up did not exert any significant influence on the concentration of Co and Cr in serum.

## Supplementary Information

Supplementary Information.

## Data Availability

The datasets generated during and/or analysed during the current study are available throughout the manuscript.
