# Supplementary material for: Serum cobalt and chromium concentration following total hip arthroplasty: a Bayesian network meta-analysis
Source: Sci Rep. 2023 Apr 27;13:6918. doi: 10.1038/s41598-023-34177-w (PMC10140036; doi:10.1038/s41598-023-34177-w)
Supplement: Supplementary file 1 — Supplementary Information. [file 41598_2023_34177_MOESM1_ESM.docx]

| **Groups** | **Difference** | **Lower CI** | **Upper CI** | **P-value** |
| --- | --- | --- | --- | --- |
| Ceramic-CoCr vs CoCr-CoCr | 1.3 | 0.736 | 1.864 | < 0.0001 |
| Ceramic-CoCr vs CoCr-Polyethylene | -0.7 | -1.2751 | -0.1249 | 0.008 |
| Ceramic-CoCr vs CoCrHC-CoCrHC | -0.2 | -0.8981 | 0.4981 | 0.9 |
| Ceramic-CoCr vs Control | -1 | -1.7163 | -0.2837 | 0.001 |
| CoCr-CoCr vs CoCr-Polyethylene | -2 | -2.3653 | -1.6347 | < 0.0001 |
| CoCr-CoCr vs CoCrHC-CoCrHC | -1.5 | -2.0386 | -0.9614 | < 0.0001 |
| CoCr-CoCr vs Control | -2.3 | -2.862 | -1.738 | < 0.0001 |
| CoCr-Polyethylene vs CoCrHC-CoCrHC | 0.5 | -0.0502 | 1.0502 | 0.09 |
| CoCr-Polyethylene vs Control | -0.3 | -0.8731 | 0.2731 | 0.6 |
| CoCrHC-CoCrHC vs Control | -0.8 | -1.4965 | -0.1035 | 0.01 |

**Appendix A:** Network comparisons of the mean concentration of Cr in serum.

| **Groups** | **Difference** | **Lower CI** | **Upper CI** | **P-value** |
| --- | --- | --- | --- | --- |
| Ceramic-CoCr vs CoCr-CoCr | 1.8 | 1.1531 | 2.4469 | < 0.0001 |
| Ceramic-CoCr vs CoCr-Polyethylene | -1.2 | -1.8597 | -0.5403 | < 0.0001 |
| Ceramic-CoCr vs CoCrHC-CoCrHC | -1 | -1.8008 | -0.1992 | 0.006 |
| Ceramic-CoCr vs Control | -1.4 | -2.2217 | -0.5783 | < 0.0001 |
| CoCr-CoCr vs CoCr-Polyethylene | -3 | -3.4191 | -2.5809 | < 0.0001 |
| CoCr-CoCr vs CoCrHC-CoCrHC | -2.8 | -3.4178 | -2.1822 | < 0.0001 |
| CoCr-CoCr vs Control | -3.2 | -3.8447 | -2.5553 | < 0.0001 |
| CoCr-Polyethylene vs CoCrHC-CoCrHC | 0.2 | -0.4312 | 0.8312 | 0.9 |
| CoCr-Polyethylene vs Control | -0.2 | -0.8575 | 0.4575 | 0.9 |
| CoCrHC-CoCrHC vs Control | -0.4 | -1.199 | 0.399 | 0.6 |

**Appendix B:** Network comparisons of the mean concentration of Co in serum.
